# Frequent deletion of the *CDKN2A* locus in chordoma: analysis of chromosomal imbalances using array comparative genomic hybridisation

**DOI:** 10.1038/sj.bjc.6604130

**Published:** 2007-12-11

**Authors:** K H Hallor, J Staaf, G Jönsson, M Heidenblad, F Vult von Steyern, H C F Bauer, M IJszenga, P C W Hogendoorn, N Mandahl, K Szuhai, F Mertens

**Affiliations:** 1 Department of Clinical Genetics, Lund University Hospital, Lund SE-221 85, Sweden; 2 Department of Oncology, Lund University Hospital, Lund SE-221 85, Sweden; 3 Department of Orthopedics, Lund University Hospital, Lund SE-221 85, Sweden; 4 Department of Orthopedics, Karolinska Hospital, Stockholm SE-171 76, Sweden; 5 Department of Molecular Cell Biology, Leiden University Medical Center, Leiden 2300 RC, The Netherlands; 6 Department of Pathology, Leiden University Medical Center, Leiden 2300 RC, The Netherlands

**Keywords:** chordoma, array CGH, *CDKN2A*, genomic imbalances

## Abstract

The initiating somatic genetic events in chordoma development have not yet been identified. Most cytogenetically investigated chordomas have displayed near-diploid or moderately hypodiploid karyotypes, with several numerical and structural rearrangements. However, no consistent structural chromosome aberration has been reported. This is the first array-based study characterising DNA copy number changes in chordoma. Array comparative genomic hybridisation (aCGH) identified copy number alterations in all samples and imbalances affecting 5 or more out of the 21 investigated tumours were seen on all chromosomes. In general, deletions were more common than gains and no high-level amplification was found, supporting previous findings of primarily losses of large chromosomal regions as an important mechanism in chordoma development. Although small imbalances were commonly found, the vast majority of these were detected in single cases; no small deletion affecting all tumours could be discerned. However, the *CDKN2A* and *CDKN2B* loci in 9p21 were homo- or heterozygously lost in 70% of the tumours, a finding corroborated by fluorescence *in situ* hybridisation, suggesting that inactivation of these genes constitute an important step in chordoma development.

Usually located along the axial skeleton, primarily in the sacrococcygeal and sphenooccipital regions, chordomas are believed to be derived from remnants of the embryonal notochord (Vujovic *et al*, 2006). These tumours are rare lesions accounting for about 1–4% of all primary bone sarcomas (Mirra *et al*, 2002). Several histological subtypes have been described; classical chordoma, which is the most common entity, chondroid chordoma, featuring regions resembling cartilage, and so-called ‘dedifferentiated’ chordoma, a rare subtype showing high-grade features. Histologically, chordomas are composed of physaliphorous cells expressing a particular low molecular weight cytokeratin pattern embedded in a mucomyxoid background (Mertens *et al*, 1994; Hazelbag *et al*, 1996; Dalpra *et al*, 1999; Scheil *et al*, 2001; Tallini *et al*, 2002). Clinically, chordomas manifest as slowly growing, locally destructive lesions with a tendency to infiltrate into adjacent tissues. Metastases are rarely encountered but because of difficulties in obtaining wide-margin resection of the primary tumour, local recurrences resulting in tissue destruction are common, eventually killing the patient.

Most cytogenetically investigated chordomas have displayed near-diploid or moderately hypodiploid karyotypes, with several numerical and structural rearrangements (Mitelman *et al*, 2007). Recurrent chromosomal aberrations in chordomas, identified using G-banding, metaphase comparative genomic hybridisation (mCGH), and fluorescence *in situ* hybridisation (FISH), include loss of the entire or parts of chromosomes 3, 4, 10, 13, and 18; loss or rearrangement of 1p and 9p; and gain of chromosome 7 (Sawyer *et al*, 2001; Scheil *et al*, 2001; Tallini *et al*, 2002; Kuzniacka *et al*, 2004; Brandal *et al*, 2005). However, neither by mCGH nor cytogenetics has any consistent structural chromosome aberration been detected. Thus, to date there is no indication that balanced or unbalanced chromosomal exchanges leading to the creation of fusion genes are important in chordoma development.

In the present study, biopsies from classical chordomas were studied by bacterial artificial chromosome (BAC) array comparative genomic hybridisation (aCGH) and/or FISH, with the purpose of detecting possible cryptic chromosomal aberrations not previously identified.

## MATERIALS AND METHODS

### Patients and materials

The present study included 30 tumour samples from 26 patients (8 women and 18 men, median age 60 years). All tumours were diagnosed as classical chordoma and located in the sacrum (*n*=20), coccyx (*n*=4), or thoracic vertebrae T11–12 (*n*=2). Eighteen of the tumours were primary lesions, ten were local recurrences, and two were metastases. Clinical information is presented in Table 1.

### Chromosome banding analysis

Fresh tumour samples were processed for G-banding analysis as previously described (Mandahl, 2001), and karyotypes were described according to the guidelines in ISCN (1995) (Mitelman, 1995).

### 32k BAC microarray

Cases 1–11 were analysed using 32k tiling microarrays containing more than 32 000 partly overlapping, individual BAC clones, generating complete coverage of the human genome. The arrays were produced at the Swegene DNA Microarray Resource Center, Department of Oncology, Lund University (http://swegene.onk.lu.se) as previously described (Jönsson *et al*, 2007), using BAC clones mapped to the hg17 genome build. Extraction, labelling, and hybridisation of genomic DNA from freshly frozen tumour biopsies, as well as pretreatment and washing of slides were performed as described previously (Heidenblad *et al*, 2006). As a control for normal copy number, a DNA pool derived from multiple healthy male donors was used (Promega, Madison, WI, USA).

### 1 Mb BAC microarray

Cases 17–26, and the respective relapse in four of these tumours, were analysed with 1 Mb microarrays containing approximately 3500 BAC clones spaced at about 1 Mb density over the genome. This BAC set is distributed to academic institutions by the Welcome Trust Sanger Institute (UK) at no cost, and information regarding the full set is available at the Sanger Center mapping database site, Ensembl (http://www.ensembl.org). The clones were spotted in triplicate, and the slides used in the current study were produced at Leiden University Medical Center, as previously described (Knijnenburg *et al*, 2005). DNA isolation, labelling, and microarray hybridisation were performed as described previously (Rozeman *et al*, 2006; Knijnenburg *et al*, 2007).

### Image and data analysis

Primary data were collected using the GenePix Pro 4.0 software (Axon Instruments Inc., Foster City, CA, USA), and the quantified data matrix was deposited into the web-based database BioArray Software Environment (BASE) (Saal *et al*, 2002). Following background correction using the median foreground minus the median background signal intensity for each channel, the log 2 ratios were calculated for each spot. Unreliable features marked in the feature extraction software, and spots not showing signal-to-noise ratios ⩾5 for both channels, were removed. Normalisation of data was performed using the popLowess algorithm (Staaf *et al*, in press), with a window size of 1% and a segmentation constant of 5. Normalised data were smoothed using a three-probe moving average window with adaptive thresholds (1% window size, scaling factor 2) (Staaf *et al*, in press), to prevent smoothing artefacts and allow detection of single outlier probes (subsequently removed). Log 2 ratios for each sample and platform were segmented using a BASE implementation of CGH-Plotter (Autio *et al*, 2003), written in R (http://www.r-project.org/). The segmentation constant, c, was set to 9. Segments less than 2 probes or 500 kb in size were removed. To facilitate cross-platform comparison, segmented data was transformed into a virtual probe set with probes spaced at 50 kb throughout the entire genome by associating each platform probe to its closest virtual probe. Copy number alterations were determined by comparing the segmented log 2 ratios to gain/loss thresholds obtained by an adaptive scaling method (Staaf *et al*, in press), using a window size of 2% and a scaling factor of 2. Segments above gain threshold were set to 1, below loss threshold as −1, and in-between as 0.

Microarray data are available at GEO (http://www.ncbi.nlm.nih.gov/geo/), using the accession number GSE9023.

### Fluorescence *in situ* hybridisation

Nine of the tumours analysed with 32k aCGH and an additional five chordomas lacking material for aCGH (cases 12–16) were analysed with FISH (Table 1) as described (Dahlén *et al*, 2003). The status of the gene *CDKN2A* (*p16*) was investigated using the commercially available LSI® p16, a probe specific for the centromere of chromosome 9 (cep 9), and whole-chromosome painting probes specific for selected chromosomes (Vysis, Downers Grove, IL, USA). Whole-chromosome painting probes were used to discriminate tumour and normal cells. To determine presence/absence of *CDKN2A*, a minimum of three tumour cells displaying concordant LSI p16 status was required.

## RESULTS

**Figure 1:**
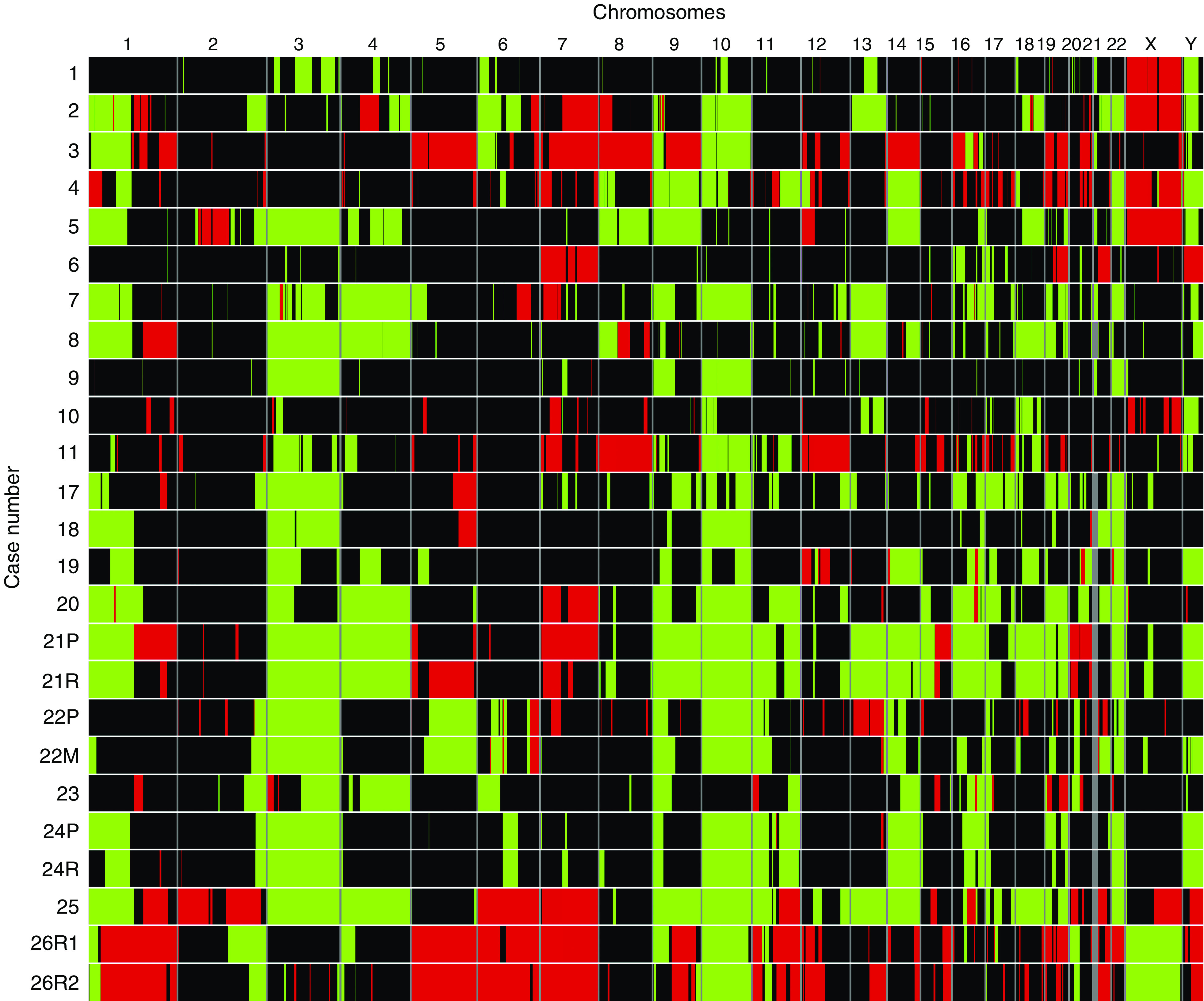
Genomic imbalances detected in individual samples. Gains (red) and losses (green) of genomic material were detected in all samples investigated by array comparative genomic hybridisation (aCGH). Each row corresponds to a separate sample and each column represents an individual chromosome. The respective relapse was investigated in four cases, and samples from the same tumour showed very similar patterns of aberrations. However, although the pattern of aberrations was almost identical, a few aberrations escaped detection in one of the samples from the same tumour. This was primarily found for low copy number changes and can most probably be explained by normal cell contamination (See online version for colour figure.).

**Figure 2:**
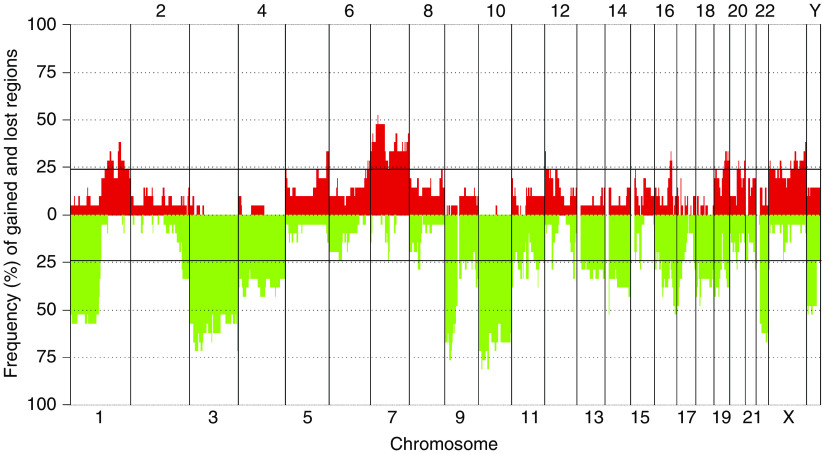
Frequency of DNA copy number changes detected by array comparative genomic hybridisation (aCGH) in 21 chordomas. Copy number alterations present in five or more of the samples were identified on all chromosomes. The number of deletions was larger than the number of gains, and the size of the deleted regions was significantly larger than the gained regions. The genomic positions of the imbalances are presented in Table 2.

The karyotypes, based on G-banding, multicolour combined binary ratio (COBRA)-FISH and DNA copy number profiles, are presented in Table 1. Five out of the nine previously unpublished karyotypes displayed a normal chromosome complement, and four showed a near-diploid chromosome number with multiple chromosomal imbalances. All cases, including the five tumours with normal G-banding karyotypes, displayed genomic imbalances upon aCGH analysis (Figure 1). Changes affecting five or more of the samples were identified on all chromosomes (Figure 2, Table 2), and in each case, one-third (median 0.33, range: 0.13–0.73) of the investigated clones showed copy number alterations. There was a median of 23 deletions resulting in, on average, loss of 678 Mb per tumour. The corresponding figures for the gained regions were significantly smaller, with a median number of 10 gained regions and a total size of 177 Mb per tumour (*P*<0.01, Mann–Whitney *U*-test). High-level amplifications were not detected in any case, and no small deletion was identified throughout all samples although recurrent narrow deletions (∼1 Mb) were found on several chromosomes.

**Figure 3:**
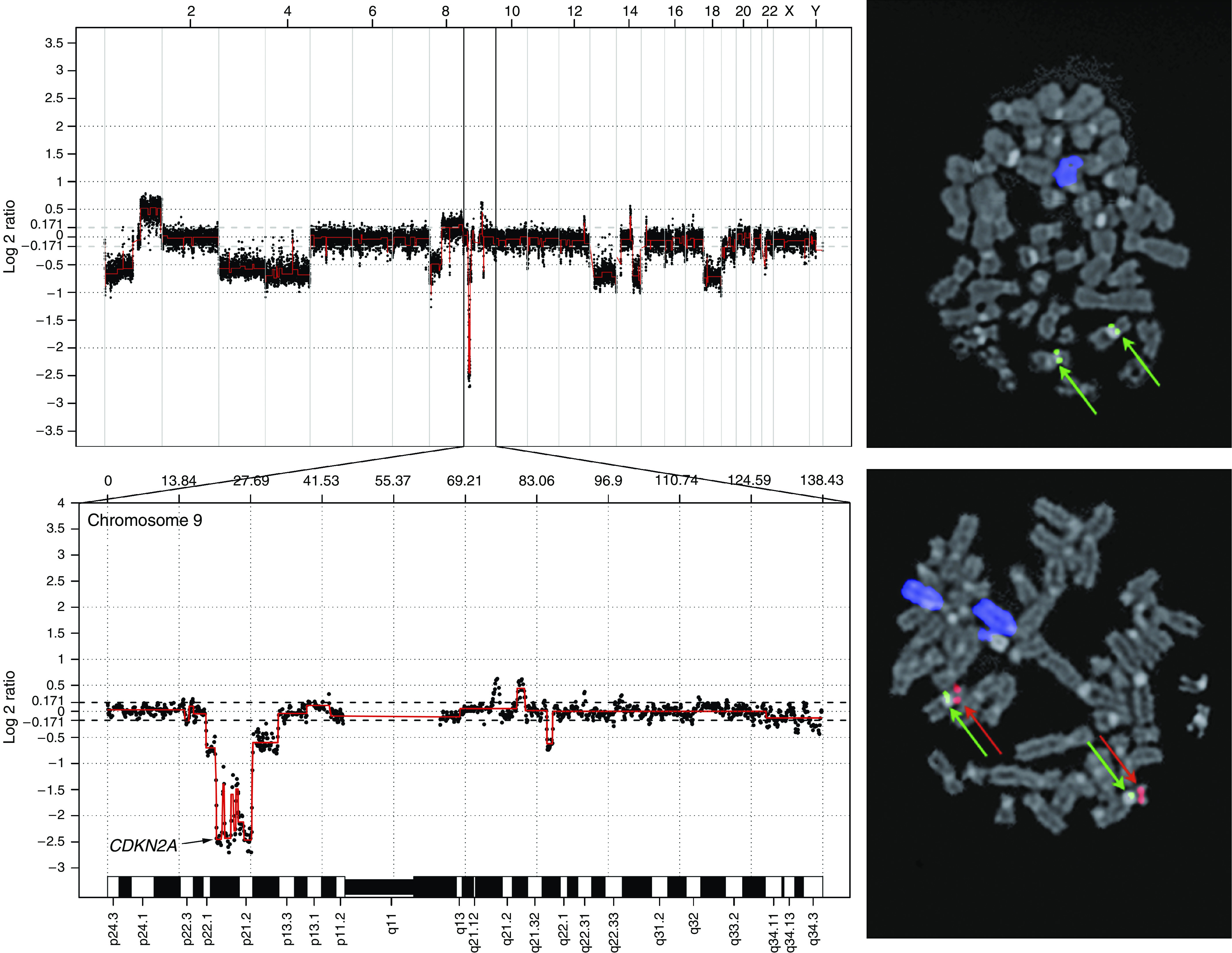
DNA copy number changes in a representative chordoma. Genomic profile of case 8 analysed using 32k array comparative genomic hybridisation (aCGH; top left). Tumour/reference log 2 ratios are displayed as the moving average of three consecutive bacterial artificial chromosome (BAC) clones, and individual chromosomes are separated by vertical bars. The profile demonstrates multiple imbalances, e.g., loss of chromosome 13 and homozygous deletion of *CDKN2A* (*p16*) on chromosome 9 (bottom left). Fluorescence *in situ* hybridisation (FISH) analysis of the same case displays loss of chromosome 13 (blue) and homozygous deletion of *CDKN2A* (*p16*) (top right). For comparison, a normal cell shows two chromosomes 13 and two normal chromosomes 9, with centromere of chromosome 9 (cep 9) and LSI p16 indicated in green and red, respectively (bottom right) (See online version for colour figure.).

Homozygous deletions were found on chromosomes 8, 9, and 18. The losses on chromosomes 8 and 18 were found in one case each, and the affected regions (7.04–7.84 and 33.45–35.04 Mb, respectively) did not harbour any obvious candidate genes. The homozygous deletions on chromosome 9 were located in the region 20.44–27.96 Mb, covering the *CDKN2A* locus in chromosomal subband 9p21.3. By aCGH, cases 4, 5, 7, 9, 11, and 19–26 showed a heterozygous deletion and cases 2, 3, and 8 displayed a homozygous loss. These findings were confirmed by FISH in nine cases (Figure 3; Table 1). In addition, this region was investigated in five samples lacking material for aCGH. Two of these showed a heterozygous deletion of LSI p16. Thus, of a total of 26 tumours investigated, 15 (58%) displayed a heterozygous deletion of the region covering the *CDKN2A* locus, and 3 (12%) showed a homozygous deletion (Table 1).

In four of the tumours, also the respective relapse was analysed with the 1 Mb microarrays. The DNA profiles of the samples from the same tumour were highly similar (Figure 1), and the relapses were excluded from further analyses.

The number and the size of the aberrations were not significantly different in the six tumours that later metastasised, compared with the rest of the tumours. Neither was there any chromosomal aberration that could be specifically linked to the group of tumours that developed metastases.

## DISCUSSION

In the present study, aberrant DNA copy number profiles were detected in 21 chordomas. Primarily losses of large chromosomal regions were found; high-level amplifications were not detected, and there was no small deletion common to all samples. However, frequent small deletions were found on several chromosomes. Whether loss of these regions results in functional inactivation of genes important in tumour development or reflects normal copy number variation remains to be elucidated.

Overall, the results were highly consistent with previous cytogenetic and mCGH findings, confirming that chordoma is a genetically heterogeneous tumour lacking apparent recurrent structural rearrangements, but demonstrating frequent imbalances of large chromosomal regions.

### Frequently deleted regions

Deletions affecting five or more samples were found on all chromosomes, except chromosome 5, and included loss of the entire or major parts of chromosome arm 1p and chromosomes 3, 4, 9, 10, 13, 14, 16, 18, 19, and 22 (Figure 2; Table 2).

Loss or rearrangement of 1p36 is a common finding in sporadic chordoma, and this region has also been associated with hereditary chordoma (Mertens *et al*, 1994; Dalpra *et al*, 1999; Miozzo *et al*, 2000; Riva *et al*, 2003; Kuzniacka *et al*, 2004). By loss of heterozygosity analysis, the 1p36.31–p36.13 region was linked to familial as well as sporadic chordoma (Miozzo *et al*, 2000), and the same group later delimited the region for sporadic chordoma development to 1p36.13 (Riva *et al*, 2003). In the present study, a minimally deleted region in 1p36.31–p36.11 was found. This region contains several genes including *RUNX3*, a transcription factor, which has been shown to be frequently deleted or transcriptionally silenced in a number of cancers, and it has been suggested to encode an important tumour suppressor (Blyth *et al*, 2005). Furthermore, this gene has been shown to be implicated in chondrocyte maturation, providing a biological link to the development of chordoma (Soung *et al*, 2007).

Frequent loss of chromosome arm 9p has previously been described in chordomas (Scheil *et al*, 2001; Kuzniacka *et al*, 2004; Brandal *et al*, 2005), and particularly, the region covering the *CDKN2A* (*p16* and *p14*) and *CDKN2B* (*p15*) loci in chromosomal band 9p21 has been shown to be deleted in many tumour types (Gil and Peters, 2006), also in chondrosarcoma (van Beerendonk *et al*, 2004). Here, we provide further evidence that loss of this region is an important event also in chordomas, with 70% of the tumours showing deletion of this region. Interestingly, in addition to the three reported cases with a homozygous deletion, three more cases in fact showed homozygous loss of the clone RP11-467K20 (cases 4, 5, and 11). This clone covers exon 1 of *CDKN2A* (isoform 4) as well as the entire *CDKN2B*, suggesting that additional deletions affecting this region would have been identified using arrays with even higher resolution. Noteworthy, the group of six patients with potential homozygous loss of *CDKN2A* and *CDKN2B* contained all five patients, investigated by aCGH, who died from their disease. Furthermore, although no particular aberration could be discerned distinguishing metastasising from nonmetastasising tumours using aCGH, deletion of this locus was found in all tumours that metastasised compared to two-thirds of the nonmetastasising tumours (data not shown). Taken together, our results are in agreement with a recent study in which immunohistochemic staining for the CDKN2A protein in chordoma consistently yielded negative results (Naka *et al*, 2005), and indicate that inactivation of CDKN2A may be important for chordoma development, although not tumour-type specific.

In this context, it could be noted that loss of heterozygosity previously has been found for the *RB1* gene in chordoma (Eisenberg *et al*, 1997), supporting a fundamental role for the RB1-signalling pathway in chordoma oncogenesis. In line with these findings, the TP53 pathway also seems to be frequently affected in chordoma; both *TP53* and *TP53BP1* were recurrently deleted. Moreover, the *CHEK2* gene is located in a region on chromosome 22, which was lost in 13 of the cases. CHEK2 is considered a tumour suppressor and mutations of *CHEK2* have been implicated in the pathogenesis of various types of familial as well as sporadic tumours, for example, the malignant bone tumour osteosarcoma (Miller *et al*, 2002). In the minimally deleted region on chromosome 11, which was lost in eight cases, *ATM* is located. The corresponding protein is believed to be important for cell response to DNA damage and for genome stability by regulating signalling pathways involving CHEK2, TP53, and a variety of additional cell cycle checkpoint proteins (Lavin and Kozlov, 2007).

Although the present study confirmed a frequent loss of chromosomes 3, 4, 10, 13, and 18, no obvious candidate tumour suppressors were found in the minimal deleted regions. Thus, either these chromosomes harbour several genes of importance for tumour development, requiring large regions to be deleted to obtain a tumourigenic effect, or the functional inactivation is preferentially achieved through large rearrangements. The same is probably true for chromosomes 14, 16, and 19, which previously have not been reported to be frequently deleted chromosomes in chordoma.

### Frequently gained regions

Generally, gains were smaller than losses and not as frequently observed. However, as shown in previous studies, gain of chromosome 7 is a common finding in chordomas (Sawyer *et al*, 2001; Scheil *et al*, 2001; Kuzniacka *et al*, 2004; Brandal *et al*, 2005). The most common gain, detected in more than half of the samples, was found in 7p15.1. This region harbours the genes *CREB5*, *CPVL*, and *CHN2*, none of which has any obvious role in chordoma development. Hence, it is likely that gain of large regions of this chromosome is required for tumour formation.

The gene expression pattern in chordoma has in a recent study been shown to cluster with cartilaginous tumours, particularly chondrosarcomas (Henderson *et al*, 2005), although the cDNA expression patterns clearly differ from other reported studies on chondrosarcoma as well as other cartilaginous tumours (Rozeman *et al*, 2005; Romeo *et al*, 2007). The study of Henderson *et al* (2005), however, suggests that genes involved in cartilage development might be of importance for chordoma oncogenesis. In line with these findings, the locus for *TGFBI* on chromosome 5 was gained in five of the cases in the present study. The corresponding protein product has been suggested to be involved in cartilage development by stimulating the growth of prechondrogenic cells (Ohno *et al*, 2002). Similarly, SOX5 is a protein believed to play an essential role in chondrocyte differentiation, and five cases displayed gain of the region on chromosome arm 12p, which harbours the *SOX5* gene (Lefebvre *et al*, 2001). Importantly, a transcription factor known as brachyury was demonstrated to be exclusively expressed in chordoma (Vujovic *et al*, 2006), which ends the long-lasting chondroid–chordoid dilemma (Romeo and Hogendoorn, 2006). The gene encoding brachyury (*T*) is located in band 6q27, and the chromosomal region covering this gene was gained in six of the cases. Furthermore, none of the samples showed deletions that could have affected this gene.

## CONCLUSIONS

The DNA copy number profiles were consistent with previous cytogenetic and mCGH findings. However, many of the DNA copy number abnormalities identified in the present study would not have been detected using mCGH, due to the small size of the imbalances. In addition, even though several of the samples analysed herein displayed a normal karyotype upon G-banding, aCGH detected chromosomal aberrations in all cases. This is most likely explained by a growth advantage *in vitro* for normal cell populations. Thus, although it has been suggested that chromosomal abnormalities in chordomas represent late events in tumour progression (Sawyer *et al*, 2001; Sandberg and Bridge, 2003), the results in the current study indicate that all chordomas harbour chromosomal imbalances.

In agreement with previous studies (Scheil *et al*, 2001), recurrent tumours did not show more chromosomal abnormalities than the respective primary lesions; in fact, the DNA profiles were almost identical. Neither did the DNA copy number pattern differ between tumours that developed metastases and the nonmetastasising tumours. Thus, although the number of cases studied was low, there was no obvious correlation between the number, size, or location of the aberrations detected and the clinicopathologic features.

We were not able to distinguish any chordoma-specific markers useful for diagnosis. In fact, many of the findings in this study, such as loss of 1p36, 9p, and 10p and gain of 7p are abnormalities previously detected in other bone tumours, for example, chondrosarcomas (Bovee *et al*, 2001; Sandberg and Bridge, 2003). Nonetheless, the characterisation of DNA copy numbers in chordoma provides important information about the genetic basis of chordoma development, and clinically important aberrations will hopefully emerge from future studies when the copy number alterations can be associated with gene expression profiles.

## Figures and Tables

**Table 1:** Clinical, cytogenetic, and FISH data

**Case no.^a^**	**Age/sex**	**Site^b^**	**Size^c^**	**Treatment^d^**	**Follow-up^e^**	**Karyotype^f^**	**LSI^®®^ p16/cep 9^g^**
1P	73/F	S	8	SI, RT	R 22, AwD 26	40-42,X,-X,-3,-4,-10,del(11)(q23),-16,-21,-22,inc	++/++
2R	37/F	S	3	SI, RT, IFN	R 97+169+274+307, M 217, DoD 338	46,XX	−−/++
3P	56/M	S	12	SI	R 26, DoD 40	46,XY	ND
4P	52/F	S	10	SM, RT	R 67, M 67, DoD 70	46,XX	ND
5P	42/M	C	5	SW	NED 120	46,XY	−+/−+
6P	41/M	C	6	SW	NED 36	46,XY	++/++
7P	71/M	C	9	S, RT	R 42+114, NED 126	46,XY,t(1;8)(q21;q24),t(2;?15)(p23;q15),add(3)(q21),t(5;7)(q33;q22)/43-48,XY,add(3)(p21),-4,-11,add(11) (q23),add(12)(q13),add(14)(q12),+21,+3mar/46-48,XY,t(1;5)(q3?2;q1?5),del(8)(p21),inv(11)(p11q23),-16, add(21)(q22),+mar^h^	−+/−+
8P	61/M	S	15	SM	R 39+62, M 88, DoD 113	40,XY,der(1)t(1;21)(p11;q11),-3,-4,-8,der(8)t(1;8)(q21;p23),add(9)(q22),del(9)(p22),-13,-14,-18,der(20)t(8;20) (q11;q13),-21,+mar/77-84,idemx2,+3,+8,+2mar^h^	−−/++
9P	74/M	C	3	S	NED 18	40-42,XY,-3,der(6)t(6;9)(q?25-27;q11-12),-8,-9,der(9)t(9;10)(p24;?) or der(9)t(9;16)(p24;?),-10,dic(12;?16) (?p1?3;?)?inv(12)(p11p13),der(21)t(8;21)(q11;p13),-22^h^	−+/−+
10P	60/F	S	6	S	NED 120	43,-X,der(X;1)(q22-24;p13),der(1)t(1;11)(p1?3;p1?3),der(1;22)(q10;q10), add(3)(p12-13),der(3)t(3;12) (p25;?p11),der(5)ins(5;19)(p15;p11p12) or der(5)ins(5;19)(p15;q11q12),der(7)t(2;7)(p15-16;q21-22) or der(7) t(2;7)(q31-32;q21-22),+der(7)t(7;13)(p15;?q14)t(1;13)(p22;q22),der(8)t(7;8) (?q32;q24),?der(10)del(10)(p11) del(10)(q22),der(11)t(11;16) (p11;q11),der(12)t(7;12)(q11;p11),?inv(12)(q13q15),-13,del(14) (q32),-16,-17,-18^h^	++/++
11P	51/M	S	8	SI	R3+60+63+86+90+113, DoD 118	42,X,-Y,der(1)t(1;3)(p31;p11-12),der(2)t(2;3)(p21;?),-3,der(3)t(2;3) (?;p12),-4,der(5)t(5;16)(q33;?p?),der(7) t(5;7)(q33;q36),+der(8)t(1;8) (?;q24),del(9)(p13),-10,del(11)(q13),dup(12)(q13q24),+del(12) (q13),-16,-18, dup(18)(q?),?add(19)(q13),der(22)t(4;22)(q11;p11)/40,X,-Y,der(1)t(1;3),der(2)t(2;3),der(2)t(2;7)(p?;?),-3, der(3)t(2;3), der(4)t(4;7)(p?;?),der(5)t(5;16),der(6)t(6;7)(q?;?),der(7)t(5;7), del(9),-10,del(11),dup(12),+del(12), der(13)t(8;13) (q?;q?),-16,der(17)t(6;17)(?;q?),der(19)t(3;19),der(22)t(4;22)/46,del(X)(q24),-Y,der(1)t(1;9) (p36;?),der(2)t(2;16)(p21;?), der(3)t(3;14)(p21;q24)t(3;16)(q11;?)t(2;16)(?;?),der(4)t(4;13)(q3?;?), der(5)t(5;16),der(6)t(6;8)(p23;?p21),?del(7)(q?),der(8)t(6;8)(?;p?),del (9),del(11),der(12)t(7;12)(q?;q24)t(5;7)(q33;?), +del(12)(q13q15), der(14)t(3;14)(q21;q24),-16,der(16)t(Y;16)(q11;p13),der(17)t (9;17)(q?;p?),?add(17)(p11), der(19)t(X;19)(?q24;p13)^h^	−+/++
12R	70/M	T12	5	S, RT	R 12+23, AwD 61	40-44,XY,-1,der(3)t(1;3)(q11;q11),?-4,der(9)t(9;14)(p11;p13),-22^h^	−+/++
13R	71/M	T11	5	SI, RT	R 6, DoD 50	47-48,XY,+2,inv(9)(p11q12)c,+13,-14,-16,-16,+2mar (primary tumour)^h^	++/++
14P	50/M	S	8	SM	NED 22	43-46,XY,-3,+12,-13,add(21)(q?21),+der(?)t(?;1)(?;q21)	++/++
15R	63/M	S	?	SW	R 157, NED 200	39-40,XY,t(1;20)(q12;q13),-3,t(6;9)(q27;q13),-10,-14,-18,-21,-22/36-40,XY,-3,t(6;9),-10,-14,-18,der(20) t(1;20),-21,-22	−+/++
16M	32/M	S	?	S, RT	R, M, DoD ∼285	33-40,X,-Y,-1,add(1)(p11)x1-2,+2,der(2;14)(q10;q10),-3,add(4)(p15),-5,-6,ins(6;?1)(q24;q25q44),-8,-9,-10, add(11)(p15),-12,der(12)t(8;12) (q13;q24),-13,-13,-15,add(16)(q22),-17,-18,-20,-21,-21,-22,+der(?) t(?;13) (?;q13),+1-4r,+4mar^h^	++/++
17R	59/M	S	?	SW	NED 64	ND	ND
18P	85/F	S	?	SI	DoC 20	ND	ND
19P	66/M	S	?	SM	AwD 48	ND	ND
20P	68/F	S	?	SW	M 48, AwD 48	ND	ND
21P	50/M	S	?	SM	R 12, AwD 98	ND	ND
21R				SW, RT		ND	ND
22P 22M	52/F	S	?	SM	M 36, AWD 48	37-40,XX,der(1)t(1;13)(p21;q33),der(2)(2pter → 2q1∷6q?∷2q?∷6p? → 6pter),der(2;17)(p10;q10),-3,-5,del(6),del(9)(p11), der(11;14)(q10;q10),der(13),-14,der(20)t(6;20)(p?;q10)	ND
23P	70/M	S	?	SI, RT	LTF	ND	ND
24P 24R	66/M	S	?	SM, SW, RT	R 36, NED 52	ND	ND
25R	57/M	S	?	SI, RT	M, LTF	ND	ND
26R1 26R2	42/F	S	?	SI, RT, SI	R 204, AwD 210	ND	ND

aCGH=array comparative genomic hybridisation; COBRA=combined binary ratio; F=female; FISH=fluorescence *in situ* hybridisation; M=male.

a P=primary tumour; R=local recurrence; M=metastasis.

b S=sacrum; C=coccyx; T=thoracal vertebra.

c Largest diameter in cm.

d SI=intralesional excision; RT=radiotherapy (postoperative); IFN=interferon; SM=marginal excision; SW=wide excision; S=surgery, not otherwise specified.

e Follow-up in months. R=local recurrence (time to local recurrence in months specified when known); AwD=alive with disease; M=metastasis (time to metastasis in months specified when known); DoD=dead of disease; NED=no evidence of disease; DoC=dead of other causes; LTF=lost to follow-up.

f Karyotypes based on G-banding, COBRA-FISH, and aCGH results. ND=not determined.

g (+) and (−) indicate presence and absence, respectively, of signals from the probe. ND=not determined.

h Karyotypes previously published (Mertens *et al*, 1994; Kuzniacka *et al*, 2004).

**Table 2:** aCGH findings in 21 chordomas

**Regions lost or gained in ⩾5 cases^a^**		**Most frequently affected region per chromosome^a^**		
**Cytogenetic location**	**Start–end (Mb)**	**Start–end (Mb)**	**Frequency**	**Examples of candidate genes**
*Copy number losses*				
1p36.33–p11.1	0.65–124.15	6.70–26.05	0.57	*RUNX3*
		60.20–67.35	0.57	
		74.85–82.85	0.57	
2q34–q37.3	212.15–242.81	214.95–242.81	0.33	
Chromosome 3	0.04–199.45	23.05–33.35	0.71	
		47.10–48.65	0.71	
Chromosome 4	0.01–191.25	0.01–1.25	0.43	
		20.20–31.50	0.43	
		89.70–108.25	0.43	
6p21.1	41.60–43.80			
7q11.22–q11.23	71.70–74.20			
8p12–p11.1	36.90–45.15	37.85–42.25	0.29	
9p24.3–q31.3	0.03–113.20	20.30–24.19	0.76	*CDKN2A*, *CDKN2B*
9q33.3–q34.3	125.90–138.39			
Chromosome 10	0.06–135.39	11.80–12.40	0.81	
11p15.5–p15.3	0.07–12.20			
11p14.3–p11.2	24.75–47.95			
11q12.2–q13.2	61.00–67.70			
11q14.3–q25	89.85–134.43	106.95–107.70	0.38	*ATM*
12p11.21–p11.1	31.25–35.35			
12q24.31	119.80–122.75	120.20–122.70	0.33	
Chromosome 13	17.92–114.12	53.05–66.55	0.33	
		77.40–89.70	0.33	
		111.95–114.12	0.33	
Chromosome 14	18.07–106.30	18.07–19.30	0.52	
15q11.2	19.78–20.32			
15q15.1–q21.1	38.70–42.90	40.60–41.15	0.29	*TP53BP1* b
16p13.3	0.01–5.00			
16p12.3–q24.3	18.15–88.70	82.40–86.10	0.52	
17p13.3–p11.1	0–22.15	0–4.85	0.48	*TP53* b
17q25.1–q21.3	69.00–78.39			
Chromosome 18	0.02–76.10	17.40–17.95	0.48	
19p13.3–p11	0.02–28.45	2.05–3.95	0.43	
19q13.11–q13.43	37.60–63.77			
20p11.21–q11.21	25.40–31.35	28.40–29.65	0.29	
21q22.2–q22.3	41.15–46.92	43.10–46.65	0.29	
Chromosome 22	14.44–49.46	38.79–40.65	0.67	*CHEK2* b
Xp22.33	0.06–2.65	0.06–2.65	0.29	
Yp11.2	7.65–8.95			
Yq11.22-q11.23	20.75–25.55			
				
*Copy number gains*				
1q21.1–q25.2	142.95–176.10			
1q31.3–q43	197.45–239.20	201.20–202.35	0.38	
5p15.33	0.07–3.70			*TERT*
5q31.1–q31.2	131.80–135.65			*TGFBI*
5q35.1–q35.3	170.60–180.73	171.50–180.73	0.33	
6q25.3-q27	157.40–170.90	158.30–161.10	0.29	
		166.05–170.75	0.29	*T*
Chromosome 7	0.04–158.62	28.05–29.90	0.52	
8q24.21–q24.22	130.55–135.15			
12p13.33–p12.1	1.55–26.10	1.65–5.55	0.33	*CCND2*^b^, *FGF6*^b^, *SOX5*^b^
12q13.11–q13.13	46.40–51.55			*COL2A1*
15q11.2	19.10–20.05			
16q21–q22.2	61.30–69.45	63.65–68.50	0.29	
19p13.3–p13.2	4.00–8.55			
19q12–q13.43	34.40–63.77	47.70–48.30	0.33	*TGFB1* b
20q11.21–q13.12	31.40–41.90	35.40–36.80	0.29	*E2F1*^b^, *SRC*
20q13.33	59.20–62.43	59.90–61.60	0.29	

aCGH=array comparative genomic hybridisation.

a Regions <500 kb are excluded.

b Genes located within the regions lost or gained in ⩾ five cases, but outside of the most frequently affected region.
